# Aetiology of acute/subacute nephritic syndrome: results from kidney biopsy registries in Japan and Europe

**DOI:** 10.1186/s12882-025-04582-6

**Published:** 2025-11-06

**Authors:** Maria Weiner, Hitoshi Sugiyama, Shinya Kaname, Rannveig Skrunes, Anna Prenner, Matija Crnogorac, Colin Geddes, Kültigin Türkmen, Kresimir Galesic, Alexander R. Rosenkranz, Yusuke Suzuki, Shouichi Fujimoto, Mårten Segelmark

**Affiliations:** 1 Department of Nephrology and Department of Health, Medicine and Caring Sciences, Linköping University, Linköping, 581 85 Sweden; 2 Department of Human Resource Development of Dialysis Therapy for Kidney Disease, Okayama University Graduate School, Okayama, Japan; 3 Department of Nephrology and Rheumatology, Kyorin University School of Medicine, Tokyo, Japan; 4 Department of Clinical Medicine, University of Bergen, Bergen, Norway; 5 Department of Medicine, Haukeland University Hospital, Bergen, Norway; 6 Division of Nephrology, Department of Internal Medicine, Medical University of Graz, Graz, Austria; 7 Department of Nephrology and Dialysis, Dubrava University Hospital, Zagreb, Croatia; 8 Glasgow Renal and Transplant Unit, Queen Elizabeth University Hospital, Glasgow, UK; 9 Division of Nephrology, Department of Internal Medicine, Necmettin Erbakan University, Meram School of Medicine, Konya, Turkey; 10 Department of Nephrology, Faculty of Medicine, Juntendo University, Tokyo, Japan; 11 Department of Medical Environment Innovation, Faculty of Medicine, University of Miyazaki, Miyazaki, Japan; 12 Department of Clinical Sciences, Lund University, Lund, Sweden; 13 Department of Nephrology, Skane University Hospital, Lund, Sweden; 14 Department of Internal Medicine, Kichijoji Asahi Hospital, Tokyo, Japan

**Keywords:** ANCA-associated vasculitis, Acute interstitial nephritis, Acute nephritic syndrome, Geographical region, IgA nephropathy, Kidney biopsy

## Abstract

**Background:**

The combination of hematuria, proteinuria and a reduced glomerular filtration rate (GFR) in patients with no previous diagnosis of chronic kidney disease is widely considered a strong indication for kidney biopsy. This study aimed to compare the frequencies of diseases leading to this symptom constellation, and to explore differences related to age and sex using data from kidney biopsy registries in Europe and Japan.

**Methods:**

Data were retrieved from national or regional kidney biopsy registries in Japan, Sweden, Norway, Scotland, Austria, Croatia and Turkey from January 1, 2007, to December 31, 2019. Patients were included if the indication for kidney biopsy was acute/subacute nephritic syndrome, which was defined as a combination of hematuria, proteinuria and reduced GFR in a patient without a prior diagnosis of chronic kidney disease. Demographic, clinical and laboratory data were collected at the time of kidney biopsy.

**Results:**

A total of 1023 patients from Europe and 2477 from Japan were included in the study. The primary cause of acute/subacute nephritic syndrome in both regions was ANCA-associated vasculitis, followed by IgA nephropathy/vasculitis and acute interstitial nephritis. The estimated GFR was higher in Europe than in Japan, at 24 and 20 ml/min/1,73 m2, respectively. The median age was 10 years younger in European patients than in Japanese patients.

**Conclusions:**

The most common underlying causes of acute/subacute nephritic syndrome are ANCA-associated vasculitis, IgA nephropathy/vasculitis and acute interstitial nephritis. This study highlights both similarities and differences in the spectrum of underlying diagnoses and clinical presentations across ages, sexes, and geographical regions in patients presenting with acute/subacute nephritic syndrome.

**Supplementary information:**

The online version contains supplementary material available at 10.1186/s12882-025-04582-6.

## Background

Kidney disease comprises a diverse group of disorders, which may present as several different clinical patterns depending on the underlying disease mechanism. Kidney biopsy is the gold standard for obtaining a definitive diagnosis, and studies have demonstrated that results from kidney biopsies can alter prognosis and therapeutic decisions in up to 50% of cases [1, 2]. However, meaningful comparisons of data on glomerular diseases across different regions are compromised by differences in biopsy practices, including variability in biopsy rates [3, 4], differences in histological classification and coding practices [5], the time period studied, the age distribution of reported cases, and the exclusion of non-glomerular diseases and secondary glomerulonephritis [4]. Acute/subacute nephritic syndrome (ANS), in this study defined as a combination of hematuria, proteinuria and a reduced glomerular filtration rate (GFR) in a patient without a prior diagnosis of chronic kidney disease, is an alarming constellation for nephrologists and is considered a strong indication for kidney biopsy. Since biopsy practices are likely to vary less in cases of ANS, it is well-suited for cross-regional comparisons. This study aimed to compare the frequencies of underlying diseases causing ANS in Japan and Europe using data from kidney biopsy registries, and to explore differences related to age and sex.

## Methods

### Data collection

Data were retrieved from national or regional kidney biopsy registries in Japan [6], Sweden [7], Norway [8], Scotland [9], Austria, Croatia [10] and Turkey [11] from January 1, 2007, to December 31, 2019. Patients were included if the indication for kidney biopsy was acute, subacute or rapidly progressive nephritic syndrome; for the present study defined as a combination of hematuria ≥ 2 on a urine dipstick or ≥ 10 erythrocytes per high-power field (hpf), albuminuria/proteinuria above the upper reference limit for the method used, and an estimated glomerular filtration rate (eGFR) < 90 ml/min/1.73 m2 in the absence of known chronic kidney disease. The distinction between acute/subacute and chronic cases was done differently, but only registries able to identify patients who met the inclusion criteria were eligible to participate. In Sweden, Croatia, and Japan, acute/subacute nephritic syndrome and/or RPGN were predefined as biopsy indications at the time of data entry to the registries. In Norway, Austria, and Turkey nephritic syndrome was prespecified, with acute/subacute cases distinguished based on concurrent coding of an acute indication, evaluation of pre-biopsy creatinine dynamics, or exclusion of patients with chronic kidney disease. In Scotland, patients were identified among those biopsied for acute kidney injury, with additional review to exclude prerenal causes, significant chronic kidney disease, or absence of hematuria and proteinuria.

Additionally, patients with missing data on serum creatinine, hematuria, or albuminuria/proteinuria were excluded from the analysis, as these variables were required to assess eligibility according to the inclusion criteria.

Demographic, clinical and laboratory data including age, sex, serum creatinine level, plasma/serum albumin level, albuminuria/proteinuria level, hematuria, ANCA status (positive/negative) and ANCA subtype (MPO-ANCA or PR3-ANCA), presence of diabetes or hypertension, use of antihypertensive drugs, and final diagnosis were collected from the time of kidney biopsy. The estimated GFR was calculated using the Modification of Diet in Renal Disease (MDRD) formula [12] for European patients and the modified MDRD [13] for Japanese patients.

For the analysis of proteinuria, patients were classified as having significant proteinuria if the albumin to creatinine ratio (ACR) was ≥ 100 mg/mmol, protein to creatinine ratio (PCR) ≥ 150 mg/mmol, 24 albumin excretion ≥ 1 g, 24 protein excretion ≥ 1,5 g or if urinary dipstick albumin was ≥3. Nephrotic syndrome was defined as plasma/serum albumin < 30 g/L and ACR/PCR > 300 mg/mmol. Patient age groups were categorized as follows: 18–44, 45–64, 65–74, and ≥75 years. The estimated GFR was categorized as follows: 60–89, 45–59, 30–44, 15–29, and < 15 ml/min/1,73 m^2^.

### Statistical analysis

Statistical analysis was performed using SPSS Statistics for Windows software (version 28.0; IBM Corp., Armonk, NY) and JMP software program (version 17, SAS Institute Inc., Cary, NC, USA). P-values < 0.05 were considered significant. Continuous data are presented as medians with interquartile ranges and categorical data are presented as percentages. Differences between groups were analysed using the Mann-Whitney test or Kruskal-Wallis test for non-parametric data, and the Chi-square test or Fisher’s exact test for categorical data. All analyses exclude missing data.

## Results

**Fig. 1:**
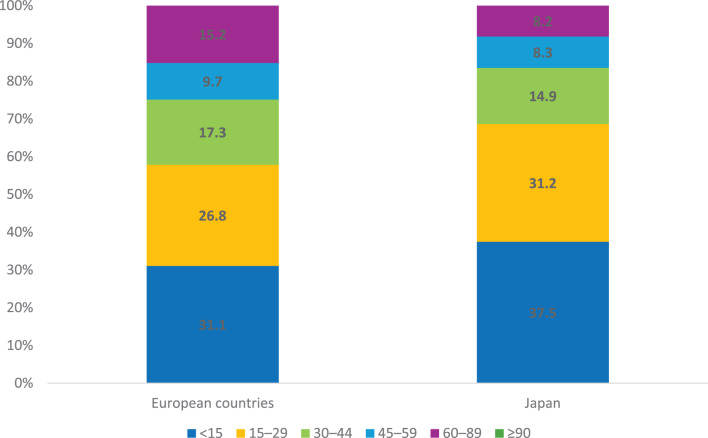
eGFR categories in all patients. Proportion of patients in different eGFR categories (ml/min/1,73 m^2^)

A total of 1023 patients from Europe and 2477 from Japan presenting with ANS were included in the study. Patient selection is shown in Supplementary Figure S1 (Additional file 1). In Europe, 59.2% of the patients were male, the median age was 58 years (IQR 41–70), and the median eGFR was 24 ml/min/1.73 m^2^ (IQR 12–44). Among the Japanese patients, 53.9% were male, the median age was 68 years (IQR 57–74), and the median eGFR at the time of kidney biopsy was 20 ml/min/1.73 m^2^ (IQR 11–35) (Table 1). The estimated GFR was higher in Europe compared to Japan (Table 1; Fig. 1). The most prevalent cause of ANS in both regions was anti-neutrophil cytoplasmic antibodies (ANCA)-associated vasculitis (AAV), accounting for 36.8% of all cases in Europe and 47.0% in Japan. This was followed by immunoglobulin A (IgA) nephropathy/vasculitis (IgAN/IgAV) at 21.7% in Europe and 11.2% in Japan, and acute interstitial nephritis (AIN) at 6.2% in Europe and 6.0% in Japan (Table 1). Demographic and clinical characteristics for the eight most common disease categories are shown in Table 2. In Europe, 1.7% of patients had post-infectious glomerulonephritis, compared with 3.4% in Japan. The most common diagnoses in patients fulfilling criteria for nephrotic syndrome in addition to acute/subacute nephritic syndrome are presented in Supplementary Table S1 (Additional file 1).

**Table 1:** Demographic and clinical data at the time of kidney biopsy

	Europe *N* = 1023	Japan *N* = 2477	*P* value
Male sex	59.2% (606)	53.9% (1336)	0.0041
Age (median)	58 (41–70)	68 (57–74)	< 0.001
BMI (median)	26 (23–29)^a^	22 (20–25)^e^	< 0.001
Creatinine (median, µmol/L)	217 (130–408)	215 (125–365)	0.032
eGFR (median, ml/min/1,73 m^2^)	24 (12–44)	20 (11–35)	< 0.001
Plasma/serum albumin (median, g/L)	32 (27–37)^b^	30 (25–36)^f^	< 0.001
Significant proteinuria	56.9% (582)	59.2% (1466)	0.21
Antihypertensive drugs	52.1% (530)^c^	52.2% (1020)^g^	0.93
Diabetes	10.6% (103)^d^	18.7% (332)^h^	< 0.001
ANCA-associated vasculitis	36.8% (376)	47.0% (1164)	< 0.001
IgA nephropathy/vasculitis	21.7% (222)	11.2% (277)	0.36
Acute interstitial nephritis	6.2% (63)	6.0% (149)	0.87
Lupus nephritis	5.3% (54)	2.0% (49)	< 0.001
Membranous nephropathy	3.2% (33)	0.7% (18)	< 0.001
Membranoproliferative glomerulonephritis	2.4% (25)	1.0% (24)	< 0.001
Anti-GBM nephritis	2.2% (23)	4.4% (110)	0.002
Acute tubular necrosis	2.3% (24)	0.4% (10)	< 0.001

BMI; body mass index, eGFR; estimated glomerular filtration rate, ANCA; anti-neutrophil cytoplasmic antibodies, IgA; immunoglobulin A, GBM; glomerular basement membrane

Differences between groups were assessed using Mann-Whitney test or χ^2^-test as appropriately

^a^Data missing for 151 patients, ^b^data missing for 22 patients, ^c^data missing for 5 patients, ^d^data missing for 55 patients, ^e^data missing for 31 patients, ^f^data missing for 14 patients, ^g^data missing for 524 patients, ^h^data missing for 702 patients

**Table 2:** Demographic and clinical data in different disease categories

Clinical characteristics Europe	Male sex	Age (median)	Creatinine (median, µmol/L)	eGFR (median, ml/min/1,73 m^2^)	Significant proteinuria	Plasma albumin (median, g/L)
ANCA-associated vasculitis (*N* = 376)	50.8% (191)	65 (55–73)	263 (168–458)	20 (10–32)	43.6% (164)	31 (27–35)
IgA nephropathy/vasculitis (*N* = 222)	71.6% (159)	42 (30–58)	144 (107–224)	40 (26–63)	67.6% (150)	35 (31–40)
Acute interstitial nephritis (*N* = 63)	61.9% (39)	56 (35–68)	420 (238–636)	12 (7–22)	36.5% (23)	32 (30–37)
Lupus nephritis (*N* = 54)	29.6% (16)	45 (31–64)	102 (82–169)	56 (32–78)	61.1% (33)	32 (27–37)
Membranous nephropathy (*N* = 33)	75.8% (25)	63 (54–71)	105 (80–217)	62 (26–78)	87.9% (29)	25 (19–32)
Membranoproliferative glomerulonephritis (*N* = 25)	76.0% (19)	63 (46–72)	189 (132–310)	30 (18–49)	80.0% (20)	30 (25–34)
Anti-GBM nephritis (*N* = 23)	43.5% (10)	56 (46–70)	647 (449–910)	6 (4–11)	52.2% (12)	28 (25–33)
Acute tubular necrosis (*N* = 24)	66.7% (16)	59 (48–70)	569 (345–842)	7 (5–17)	58.3% (14)	32 (28–37)

Clinical characteristics Japan	Male sex	Age (median)	Creatinine (median, µmol/L)	eGFR (median, ml/min/1,73 m2)	Significant proteinuria	Serum albumin (median, g/L)
ANCA-associated vasculitis (*N* = 1164)	50.1% (583)	71 (64–76)	212 (127–347)	20 (11–33)	54.0% (628)	29 (24–34)
IgA nephropathy/vasculitis (*N* = 277)	65.3% (181)	62 (46–70)	159 (97–261)	28 (16–49)	68.2% (189)	34 (28–38)
Acute interstitial nephritis (*N* = 149)	47.7% (71)	67 (57–74)	256 (176–409)	16 (10–24)	35.6% (53)	35 (31–39)
Lupus nephritis (*N* = 49)	24.5% (12)	44 (36–61)	168 (88–320)	28 (11–58)	85.7% (42)	26 (20–30)
Membranous nephropathy (*N* = 18)	55.6% (10)	72 (63–77)	185 (75–406)	25 (10–65)	94.4% (17)	29 (21–33)
Membranoproliferative glomerulonephritis (*N* = 24)	70.8% (17)	69 (64–77)	250 (135–366)	19 (11–35)	75.0% (18)	28 (25–32)
Anti-GBM nephritis (*N* = 110)	46.4% (51)	66 (55–72)	532 (276–894)	7 (4–15)	69.1% (76)	27 (23–32)
Acute tubular necrosis (*N* = 10)	70.0% (7)	68 (64–73)	487 (114–703)	10 (5–42)	70.0% (7)	30 (26–34)

ANCA; anti-neutrophil cytoplasmic antibodies, IgA; immunoglobulin A, GBM; glomerular basement membrane, eGFR; estimated glomerular filtration rate

### Sex

ANCA-associated vasculitis was more frequently the cause of ANS in women than in men; 44.4% versus 31.5% (*p* < 0.001) in Europe and 50.9% versus 43.6% (*p* < 0.001) in Japan. Conversely, IgAN/IgAV was more commonly the underlying diagnosis in men: 26.2% compared with 15.1% (*p* < 0.001) in Europe and 13.5% compared with 8.4% (*p* < 0.001) in Japan. No sex differences were observed for acute interstitial nephritis or anti-GBM nephritis. Lupus nephritis was more prevalent among females in both regions (Table 3).

**Table 3:** Demographic and clinical data in males and females

Europe	Male *N* = 606	Female *N* = 417	*P* value
Age (median)	57 (40–70)	60 (44–70)	0.19
BMI (median)	26 (23–29)	26 (23–29)	0.074
Creatinine (median, µmol/L)	229 (139–415)	198 (123–397)	0.04
eGFR (median, ml/min/1,73 m2)	25 (13–48)	23 (10–40)	0.015
Plasma albumin (median, g/L)	32 (28–37)	31 (27–36)	0.11
Significant proteinuria	59.2% (359)	53.5% (223)	0.067
Antihypertensive drugs	53.1% (321)	50.6% (209)	0.44
Diabetes	12.0% (69)	8.6% (34)	0.089
ANCA-associated vasculitis	31.5% (191)	44.4% (185)	< 0.001
IgA nephropathy/vasculitis	26.2% (159)	15.1% (63)	< 0.001
Acute interstitial nephritis	6.4% (39)	5.8% (24)	0.66
Lupus nephritis	2.6% (16)	9.1% (38)	< 0.001
Membranous nephropathy	4.1% (25)	1.9% (8)	0.050
Membranoproliferative glomerulonephritis	3.1% (19)	1.4% (6)	0.084
Anti-GBM nephritis	1.7% (10)	3.1% (13)	0.12
Acute tubular necrosis	2.6% (16)	1.9% (8)	0.45

Japan	Male *N* = 1336	Female *N* = 1141	*P* value
Age (median)	67 (58–74)	68 (56–75)	0.39
BMI (median)	22 (20–25)	22 (20–25)	< 0.001
Creatinine (median, µmol/L)	239 (142–401)	186 (107–324)	< 0.001
eGFR (median, ml/min/1,73 m2)	20 (11–35)	19 (10–35)	0.30
Serum albumin (median, g/L)	30 (25–35)	31 (26–36)	0.0058
Significant proteinuria	62.1% (829)	55.8% (637)	0.0017
Antihypertensive drugs	53.7% (567)	50.5% (453)	0.16
Diabetes	20.5% (195)	16.6% (137)	0.037
ANCA-associated vasculitis	43.6% (583)	50.9% (581)	<0.001
IgA nephropathy/vasculitis	13.5% (181)	8.4% (96)	< 0.001
Acute interstitial nephritis	5.3% (71)	6.8% (78)	0.11
Lupus nephritis	0.9% (12)	3.2% (37)	< 0.001
Membranous nephropathy	0.7% (10)	0.7% (8)	0.89
Membranoproliferative glomerulonephritis	1.3% (17)	0.6% (7)	0.095
Anti-GBM nephritis	3.8% (51)	5.2% (59)	0.10
Acute tubular necrosis	0.5% (7)	0.3% (3)	0.31

BMI; body mass index, eGFR; estimated glomerular filtration rate, ANCA; anti-neutrophil cytoplasmic antibodies, IgA; immunoglobulin A, GBM; glomerular basement membrane

Differences between groups were assessed using Mann-Whitney test or χ^2^-test as appropriately

### Age

The largest age group with ANS in Europe was the 45–64 years group, whereas in Japan, it was the 65–74 years group. Kidney function was lower at the time of biopsy in the older age groups compared to the younger age groups. The median eGFR was 42 ml/min/1.73 m^2^ in patients aged 18–44 years compared to 17 ml/min/1.73 m^2^ in patients aged 75 years or above (*p* < 0.001) in Europe. The corresponding numbers for Japan were eGFR 31 ml/min/1.73 m^2^ and 17 ml/min/1.73 m^2^ (*p* < 0.001). AAV was observed more frequently in patients of older age in both regions, while the opposite was found for IgAN/IgAV and lupus nephritis. The use of anti-hypertensive drugs and the presence of diabetes mellitus increased with age (Table 4).

**Table 4:** Demographic and clinical data in different age groups

Europe	18–44 years *N* = 293	45–64 years *N* = 352	65–74 years *N* = 235	≥75 years *N* = 144	*P* value
Male sex	62.3% (182)	59.4% (209)	56.2% (132)	57.6% (83)	0.53
BMI (median)	25 (22–29)	27 (24–31)	27 (24–29)	25 (22–27)	< 0.001
Creatinine (median, µmol/L)	152 (103–283)	220 (137–436)	265 (153–479)	286 (194–473)	< 0.001
eGFR (median, ml/min/1,73 m2)	42 (21–66)	24 (10–40)	18 (10–33)	17 (2–28)	< 0.001
Plasma albumin (median, g/L)	35 (30–40)	32 (27–36)	31 (26–36)	28 (23–33)	< 0.001
Antihypertensive drugs	35.7% (104)	54.2% (189)	63.2% (148)	61.8% (89)	< 0.001
Diabetes	3.7% (10)	10.7% (36)	15.7% (35)	16.3% (22)	< 0.001
Proteinuria	59.9% (175)	61.1% (215)	48.9% (115)	53.5% (77)	0.016
ANCA-associated vasculitis	13.0% (38)	41.8% (147)	50.2% (118)	50.7% (73)	< 0.001
IgA nephropathy/vasculitis	41.3% (121)	17.9% (63)	11.1% (26)	8.3% (12)	< 0.001
Acute interstitial nephritis	8.9% (26)	4.3% (15)	6.8% (16)	4.2% (6)	0.067
Lupus nephritis	8.5% (25)	4.5% (16)	3.8% (9)	2.8% (4)	0.036
Membranous nephropathy	1.4% (4)	4.3% (15)	3.8% (9)	3.5% (5)	0.14
Membranoproliferative glomerulonephritis	2.0% (6)	2.6% (9)	2.1% (5)	3.5% (5)	0.79
Anti-GBM nephritis	1.7% (5)	2.6% (9)	2.6% (6)	2.1% (3)	0.88
Acute tubular necrosis	1.7% (5)	3.1% (11)	3.0% (7)	0.7% (1)	0.32

Japan	18–44 years *N* = 306	45–64 years N = 698	65–74 years *N* = 864	≥75 years *N* = 609	*P* value
Male sex	46.1% (141)	57.9% (404)	55.9% (483)	50.6% (308)	0.0011
BMI (median)	23 (20–26)	22 (20–25)	22 (20–25)	22 (20–24)	<0.0001
Creatinine (median, µmol/L)	153 (88–358)	216 (117–383)	221 (135–365)	227 (141–358)	< 0.001
eGFR (median, ml/min/1,73 m2)	31 (14–60)	20 (11–39)	19 (11–32)	17 (10–30)	< 0.001
Serum albumin (median, g/L)	34 (28–38)	31 (26–36)	30 (25–35)	28 (23–33)	< 0.001
Anti-hypertensive drugs	34.3% (81)	47.5% (261)	56.4% (394)	60.4% (284)	< 0.001
Diabetes	8.8% (20)	16.6% (81)	21.1% (133)	22.8% (98)	< 0.001
Proteinuria	62.4% (191)	60.9% (425)	56.9% (492)	58.8% (358)	0.26
ANCA-associated vasculitis	13.1% (40)	38.1% (266)	56.1% (485)	61.2% (373)	< 0.001
IgA nephropathy/vasculitis	21.9% (67)	14.2% (99)	7.5% (65)	7.6% (46)	< 0.001
Acute interstitial nephritis	6.9% (21)	6.6% (46)	5.9% (51)	5.1% (31)	0.63
Lupus nephritis	8.2% (25)	2.4% (17)	0.2% (2)	0.8% (5)	< 0.001
Membranous nephropathy	0.3% (1)	0.7% (5)	0.5% (4)	1.3% (8)	0.22
Membranoproliferative glomerulonephritis	0.7% (2)	0.7% (5)	1.0% (9)	1.3% (8)	0.66
Anti-GBM nephritis	3.3% (10)	6.3% (44)	4.5% (39)	2.8% (17)	0.014
Acute tubular necrosis	0.0% (0)	0.3% (2)	0.7% (6)	0.3% (2)	0.34

BMI; body mass index, eGFR; estimated glomerular filtration rate, ANCA; anti-neutrophil cytoplasmic antibodies, IgA; immunoglobulin A, GBM; glomerular basement membrane

Differences between groups were assessed using Kruskal-Wallis test or χ^2^-test as appropriately

### ANCA-associated vasculitis

**Fig. 2:**
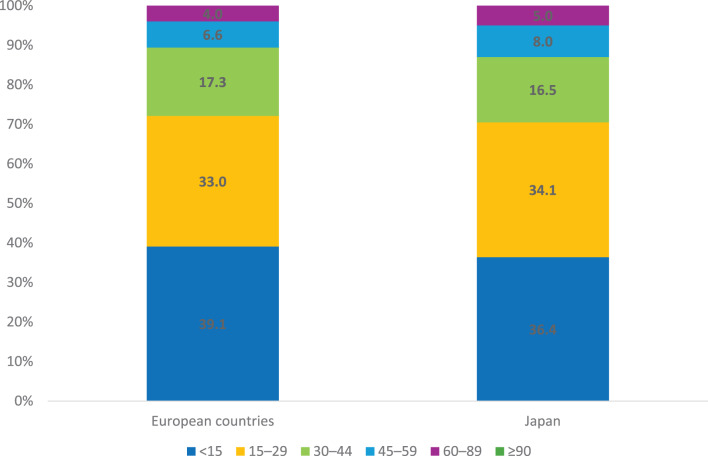
eGFR categories in ANCA-associated vasculitis. Proportion of patients in different eGFR categories (ml/min/1,73 m^2^) in ANCA-associated vasculitis

Among the patients with ANS diagnosed with AAV in Europe, 55.1% were MPO-ANCA positive and 39.4% were PR3-ANCA positive (3.8% were ANCA-negative and 1.7% double-positive). In Japan, 95.1% of patients were MPO-ANCA positive, and 4.9% were PR3-ANCA positive. The median eGFR at the time of biopsy was similar in patients with AAV in Europe and Japan (Table 2; Fig. 2). Japanese patients with AAV were significantly older than European patients, with median ages of 71 years compared with 65 years (*p* < 0.001; Table 2). MPO-ANCA-positive patients were older compared to PR3-ANCA-positive patients in both regions (68 versus 62 years in Europe and 71 versus 65 years in Japan), and a male predominance was observed among the PR3-ANCA-positive patients (60.2% and 67.3% males in Europe and Japan, respectively) (Supplementary Table S2; Additional file 1).

### IgA nephropathy/vasculitis

**Fig. 3:**
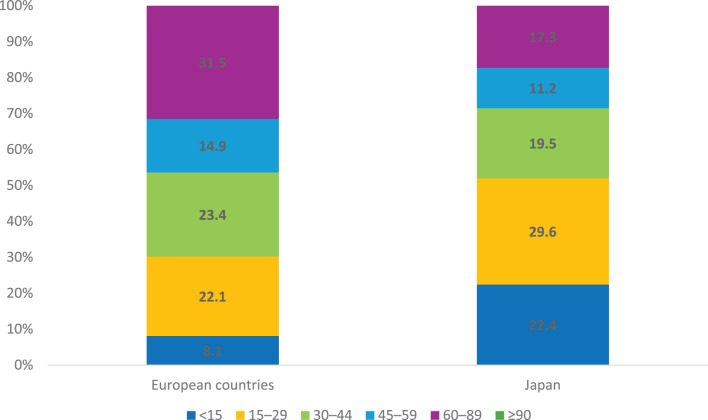
eGFR categories in IgA nephropathy/vasculitis. Proportion of patients in different eGFR categories (ml/min/1,73 m^2^) in IgA nephropathy/vasculitis

The median age of Japanese patients diagnosed with IgAN/IgAV was 62 years, compared to 42 years for European patients. Kidney impairment was more severe in Japan than in Europe, with a median eGFR of 28 ml/min/1,73 m^2^ compared with 40 ml/min/1,73 m^2^ (Table 2), and a greater proportion of patients with eGFR < 30 ml/min/1,73 m^2^ (Fig. 3). The proportion of patients with IgAV was greater in Japan (28.9%) than in Europe (13.4%). The median eGFRs for IgAN and IgAV were similar in Europe, but in Japan it was 26 ml/min/1,73 m^2^ for IgAN and 41 ml/min/1,73 m^2^ for IgAV (Supplementary Table S3; Additional file 1). The predominant age group for IgAN patients in Europe was 18–44 years, whereas IgAV was most frequently observed in patients older than 65 years. In Japan, both IgAN and IgAV were most frequently diagnosed in the age group 45–64 years (Supplementary Table S4; Additional file 1).

## Discussion

We describe data from a comprehensive multicenter register study of patients from Europe and Japan presenting with acute/subacute nephritic syndrome, highlighting both similarities and differences in disease spectrum, clinical and demographic characteristics across ages, sexes, and geographical regions. Numerous studies have compared the frequencies of kidney diseases using data from kidney biopsy registries [4]. However, such studies are often compromised by the fact that differences in biopsy frequencies and biopsy indications overshadow true epidemiological differences based on environmental and genetic factors. To reduce this bias, we limited the analysis to a restricted indication for kidney biopsy, where there is broad consensus that a biopsy is urgently needed.

The predominant underlying condition identified in both regions was AAV, followed by IgAN/IgAV and AIN. In contrast, previous data from the J-RBR reported IgAN as the most common diagnosis in patients biopsied for nephritic syndrome [14]. Similarly, data from the Polish Registry of Renal Biopsies found IgAN to be the leading cause in such patients, with pauci-immune glomerulonephritis as the second most common diagnosis [15]. Findings from the nationwide Czech Registry of Renal Biopsies also support this pattern: nephritic syndrome was present in 26% of all cases, with IgAN and necrotizing vasculitis being the predominant diagnoses, whereas AIN was relatively uncommon [16]. A plausible explanation for the discrepancy between our findings and those of other studies lies in differences in the definition of nephritic syndrome used. Previous studies did not differentiate between acute/subacute and chronic nephritic syndrome, which likely accounts for the higher frequency of IgAN.

Data from other biopsy registries have found that 50–75% of patients with systemic vasculitis, including AAV, present with nephritic syndrome [16, 17]. The severity of kidney impairment at the time of biopsy did not differ between Japanese and European patients with AAV. Our finding of a striking predominance of MPO-ANCA positivity in Japan aligns with previous studies comparing AAV in Europe and Japan [18]. These differences may reflect underlying genetic predispositions or environmental triggers that vary between populations. Genome-wide association studies (GWAS) have revealed genetic differences between MPO-ANCA positive vasculitis and PR3-ANCA positive vasculitis [19], and the prevalence of these genetic variants may differ between Europe and Japan [20].

IgAN was the second most common diagnosis in our material, but is the most prevalent primary glomerulonephritis in several studies from both national and regional kidney biopsy registries, as well as in single center studies [6, 14, 16, 21–31]. It covers a broad disease spectrum from asymptomatic urinary abnormalities to rapidly progressive glomerulonephritis. In a study from Turkey on primary glomerular diseases, 35.5% of patients with IgAN presented with nephritic syndrome [21]. Similar numbers were observed in Brazil [17] and in the Czech Republic [16]. There were significant differences in both age and eGFR between Europe and Japan, with the Japanese patients being older and presenting with more severe kidney impairment. More patients were also diagnosed with IgAV among the Japanese patients. In the Japan Renal Biopsy Registry (J-RBR), chronic nephritic syndrome was more common in IgAN, whereas acute and rapidly progressive nephritic syndrome with histologically confirmed endocapillary proliferation and crescentic glomerulonephritis was more common in IgAV [32]. Previous studies comparing IgAN in different geographic regions and different ethnic groups have found large differences in clinical presentation, histopathological findings, and outcomes. Biopsy studies have found differences in the frequency of the pathological patterns included in the MEST-C score, with endocapillary hypercellularity and crescents being more common in Japan compared to Europe [33].

Acute interstitial nephritis was the third most common diagnosis in the present study but accounted for only 6% of all cases of ANS in both Europe and Japan. In the Czech Registry of Renal Biopsies, AIN was found in 3.3% of all biopsies performed with 24.7% presenting with the nephritic syndrome [16]. In Romania, 40% of patients with biopsy-verified AIN presented with nephritic syndrome [34]. The definition of ANS in this study was based on the clinical setting, without knowledge of whether the underlying condition was of glomerular or non-glomerular origin. Accordingly, patients later found to have non-glomerular diseases, such as AIN, were not excluded. We consider this approach clinically relevant, as it reflects real-life practice where it is not possible to distinguish between these entities at initial patient evaluation. A definitive diagnosis can be established only after kidney biopsy, when the clinical presentation is integrated with histopathological findings.

A small subset of patients also met criteria for nephrotic syndrome, presenting with a mixed nephrotic–nephritic picture. Within this group, AAV and IgAN/IgAV remained the most common diagnoses. Membranous nephropathy was observed more frequently in this group compared to the overall study population. Microscopic hematuria is not uncommon in membranous nephropathy [7], and such patients may present with a clinical picture consistent with ANS. However, the concomitant presence of marked proteinuria and hypoalbuminemia likely reinforce the indication for kidney biopsy.

The median age of the European patients was 10 years younger than that of the Japanese patients. This could reflect differences in biopsy practices, a possible greater inclination to perform kidney biopsies in patients of advancing age in Japan, or differences in the disease spectrum between the studied regions. It should also be noted that the median age of the overall population in Japan is greater than in most European countries [35]. An increasing age at the time of biopsy and a larger proportion of patients > 65 years undergoing kidney biopsy have been described from different regions [16, 25, 26, 28, 29], although this trend has not been observed in all studies [34]. As shown in previous studies, AAV was more prevalent in older age groups, whereas IgAN/IgAV was more frequently found in younger patients [17, 21, 36]. Accordingly, the higher age of patients in this study compared to others could be one of the factors contributing to the greater frequency of AAV reported. Japanese patients with AAV were older than their European counterparts, which may also be attributable to the predominance of MPO-ANCA–positive AAV in Japan, as patients with MPO-ANCA positivity are typically older than those with PR3-ANCA positivity [37].

There was a male predominance overall. Previous studies from biopsy registries have found a male predominance in North America and Europe, but a female predominance in Latin America and an equal distribution in Asia [38]. AAV was more often the underlying cause of nephritic syndrome among women than among men, although there was an equal sex distribution among the AAV-patients overall. Previous studies have reported that the sex distribution in AAV is fairly equal, but a male predominance has been reported for granulomatosis with polyangiitis (GPA), and a female predominance for microscopic polyangiitis (MPA), although this is not apparent across all studied regions [39]. In the present study, male sex was more common among PR3-ANCA positive patients. IgAN/IgAV exhibited a male predominance in both regions, which has been reported previously [3, 6, 16, 24–27, 30, 34, 36, 40].

In this study, we employed the term acute/subacute nephritic syndrome to describe the clinical presentation of hematuria, proteinuria, and reduced GFR in a patient with no prior diagnosis of kidney disease. To harmonize with the concept of acute kidney disease (AKD) proposed in a Kidney Disease: Improving Global Outcomes (KDIGO) consensus conference [41], an even better term would be simply acute nephritic syndrome. The AKD concept emphasizes that many patients present with structural and/or functional kidney changes, such as those seen in ANS, that fall outside the traditional definitions of acute kidney injury (AKI) and chronic kidney disease (CKD) but still warrant rapid and thorough diagnostic evaluation. The importance of identifying patients who present with this clinical syndrome is underscored by the findings of this study: more than 40% of the patients received a diagnosis of AAV following biopsy, a condition that requires prompt recognition and treatment.

Acute/subacute nephritic syndrome encompasses most cases that in other studies would be referred to as rapidly progressive glomerulonephritis (RPGN), but also cases with post-infectious glomerulonephritis. The term RPGN, usually defined as doubling of the serum creatinine level within three months, requires both an observational period and histological confirmation. Even more troublesome for epidemiological studies, some authors use RPGN as a synonym of diffuse crescentic glomerulonephritis, which excludes all patients with a diagnosis based mainly on clinical features and serology. Some authors include hypertension as a component of the acute nephritic syndrome [42, 43]. However, many patients with proteinuria, hematuria, and deterioration of GFR have normal blood pressure at presentation. A recent study from India found that only 60% of patients with glomerulonephritis due to infections had hypertension at diagnosis, regardless of whether it was peri-, para- or post-infectious glomerulonephritis [44]. In the present study, hypertension was not included in the definition of ANS; however, we found that approximately 50% of patients were receiving antihypertensive medication at the time of kidney biopsy. Unfortunately, data were not available to determine whether the hypertension was chronic or newly diagnosed.

The strengths of this study include the large number of patients from several kidney biopsy registries. The three most common diagnoses were similar in Europe and Japan, increasing the applicability of the results across geographical regions. All participating centers were required to identify patients who fulfilled our predefined inclusion criteria. However, differences in biopsy practices between centers may exist. Consequently, despite applying the same inclusion criteria, we may not have fully captured the same clinical indication for kidney biopsy among all patients included. Given the nature of the registry data, it was not possible to make a distinction between primary and secondary glomerulonephritis or to verify whether the diagnoses were based on both clinical and histopathological data, as this would have required access to patient chart review. Likewise, we did not have the possibility to ascertain if historic data on urinalysis and eGFR measurements were available.

## Conclusions

In summary, the predominant underlying diagnoses in patients presenting with acute/subacute nephritic syndrome in both Europe and Japan are AAV, IgAN/IgAV and AIN. The clinical triad of hematuria, proteinuria and reduced eGFR represents a strong indication for kidney biopsy, where underlying conditions necessitating urgent management may be identified. Knowledge of similarities and differences between regions can aid in our understanding of how disease patterns are influenced by environmental and genetic factors, as well as factors related to the healthcare system.

## Electronic supplementary material

Below is the link to the electronic supplementary material.

Supplementary Material 1

Supplementary Material 2

## Data Availability

Data from European patients are available from the corresponding author upon reasonable request. Data from Japanese patients are not available for public review.

## Abbreviations

ACR: Albumin to creatinine ratio
AAV: ANCA-associated vasculitis
AIN: Acute interstitial nephritis
AKD: Acute kidney disease
AKI: Acute kidney injury
ANCA: Anti-neutrophil cytoplasmic antibodies
ANS: Acute/subacute nephritic syndrome
BMI: Body mass index
CKD: Chronic kidney disease
eGFR: Estimated glomerular filtration rate
ERA: European Renal Association
GBM: Glomerular basement membrane
GPA: Granulomatosis with polyangiitis
GWAS: Genome-wide association studies
IgA: Immunoglobulin A
IgAN: IgA nephropathy
IgAV: IgA vasculitis
J-RBR: Japan Renal Biopsy Registry
KDIGO: Kidney Disease: Improving Global Outcomes
MDRD: Modification of Diet in Renal Disease
MPA: Microscopic polyangiitis
PCR: Protein to creatinine ratio
PR3: Proteinase-3
RPGN: Rapidly progressive glomerulonephritis
