# Supplementary material for: Aetiology of acute/subacute nephritic syndrome: results from kidney biopsy registries in Japan and Europe
Source: BMC Nephrol. 2025 Nov 6;26:625. doi: 10.1186/s12882-025-04582-6 (PMC12593942; doi:10.1186/s12882-025-04582-6)
Supplement: Supplementary file 1 — Supplementary Material 1 [file 12882_2025_4582_MOESM1_ESM.docx]

**Supplementary Figure S1.** Selection of patients in the study.

**Supplementary Table S1.** Diagnoses in patients with mixed nephrotic-nephritic syndrome.

|  | Europe (N=128) | Japan (N=346) |
| --- | --- | --- |
| ANCA-associated vasculitis | 17.2% (22) | 27.7% (96) |
| IgA nephropathy/vasculitis | 14.1% (18) | 15.3% (53) |
| Acute interstitial nephritis | 2.3% (3) | 2.0% (7) |
| Lupus nephritis | 7.0% (9) | 6.9% (24) |
| Membranous nephropathy | 13.3% (17) | 2.3% (8) |
| Membranoproliferative glomerulonephritis | 5.5% (7) | 2.6% (9) |
| Anti-GBM nephritis | 5.5% (7) | 4.3% (15) |
| Acute tubular necrosis | 1.6% (2) | 0.6% (2) |

ANCA; anti-neutrophil cytoplasmic antibodies, IgA; immunoglobulin A, GBM; glomerular basement membrane

**Supplementary Table S2.** Demographic and clinical data in ANCA subtypes

| Europe | Male sex | Age (median) | Creatinine (median, µmol/L) | eGFR (median, ml/min/1,73 m2) | Significant proteinuria | Plasma albumin (median, g/L) |
| --- | --- | --- | --- | --- | --- | --- |
| MPO-ANCA | 44.3% (70) | 68 (57-74) | 224 (152-362) | 23.0 (11.8-34.0) | 44.9% (71) | 32 (28-37) |
| PR3-ANCA | 60.2% (68) | 62 (50-73) | 240 (150-420) | 20.0 (12.0-38.0) | 32.7% (37) | 30 (23-33) |
| Double-positive | 20.0% (1) | 70 (55-72) | 200 (155-463) | 22.0 (13.5-31.0) | 40.0% (2) | 30 (27-32) |
| Negative | 45.5% (5) | 67 (63-71) | 243 (199-289) | 20.0 (15.0-25.0) | 45.5% (5) | 29 (25-31) |

| Japan | Male sex | Age (median) | Creatinine (median, µmol/L) | eGFR (median, ml/min/1,73 m2) | Significant proteinuria | Serum albumin (median, g/L) |
| --- | --- | --- | --- | --- | --- | --- |
| MPO-ANCA | 49.2% (546) | 71 (64–76) | 212 (127–352) | 19.4 (11.2–32.6) | 54.0% (599) | 29 (24–34) |
| PR3-ANCA | 67.3% (37) | 65 (55–72) | 172 (105–277) | 27.6 (14.8–43.9) | 52.7% (29) | 26 (23–33) |
| Double-positive | Not surveyed | | | | | |
| Negative |  |  |  |  |  |  |

MPO; myeloperoxidase, ANCA; anti-neutrophil cytoplasmic antibodies, PR3; proteinase-3, eGFR; estimated glomerular filtration rate

**Supplementary Table S3.** Demographic and clinical data in IgA nephropathy/vasculitis

| Europe | Male sex | Age (median) | Creatinine (median, µmol/L) | eGFR (median, ml/min/1,73 m2) | Significant proteinuria | Plasma albumin (median, g/L) |
| --- | --- | --- | --- | --- | --- | --- |
| IgA nephropathy | 74.8% (116) | 37 (28-53) | 153 (113-245) | 39.0 (24.0-62.0) | 65.2% (101) | 35 (31-40) |
| IgA vasculitis | 58.3% (14) | 68 (59-76) | 128 (102-198) | 38.5 (27.3-61.8) | 75.0% (18) | 30 (25-39) |

| Japan | Male sex | Age (median) | Creatinine (median, µmol/L) | eGFR (median, ml/min/1,73 m2) | Significant proteinuria | Serum albumin (median, g/L) |
| --- | --- | --- | --- | --- | --- | --- |
| IgA nephropathy | 66.0% (130) | 60 (44–70) | 177 (119–300) | 26.1 (14.4–41.8) | 69.5% (137) | 34 (29–38) |
| IgA vasculitis | 63.8% (51) | 63 (49–73) | 109 (79–218) | 40.6 (20.5–68.6) | 65.0% (52) | 31 (27–37) |

IgA; immunoglobulin A, eGFR; estimated glomerular filtration rate

**Supplementary Table S4.** Demographic data by age groups in IgA nephropathy/ vasculitis

| Europe | 18-44 years  N=96 | 45-64 years  N=50 | 65-74 years  N=22 | ≥75 years  N=11 |
| --- | --- | --- | --- | --- |
| IgA nephropathy | 59.4% (92) | 29.7% (46) | 8.4% (13) | 2.6% (4) |
| IgA vasculitis | 16.7% (4) | 16.7% (4) | 37.5% (9) | 29.2% (7) |

| Japan | 18-44 years  N=67 | 45-64 years  N=99 | 65-74 years  N=65 | ≥75 years  N=46 |
| --- | --- | --- | --- | --- |
| IgA nephropathy | 26.4% (52) | 36.0% (71) | 22.8% (45) | 14.7% (29) |
| IgA vasculitis | 18.8% (15) | 35.0% (28) | 25.0% (20) | 21.3% (17) |

IgA; immunoglobulin A
